# Role of parental and environmental characteristics in toddlers’ physical activity and screen time: Bayesian analysis of structural equation models

**DOI:** 10.1186/s12966-018-0649-5

**Published:** 2018-02-09

**Authors:** Eun-Young Lee, Kylie D. Hesketh, Ryan E. Rhodes, Christina M. Rinaldi, John C. Spence, Valerie Carson

**Affiliations:** 1 Faculty of Kinesiology, Sport, and Recreation, University of Alberta, Edmonton, AB T6G 2H9 Canada; 2 Institute for Physical Activity and Nutrition, School of Exercise and Nutrition Sciences, Faculty of Health, Deakin University, Geelong, VIC 3220 Australia; 3 School of Exercise Science, Physical and Health Education, University of Victoria, Victoria, BC V8W 2Y2 Canada; 4 Department of Educational Psychology, Faculty of Education, University of Alberta, Edmonton, AB T6G 2G5 Canada

**Keywords:** Screen time limits, Parental modeling, Barrier self-efficacy, Outcome expectations, Bayesian theorem, Socialization model of child behavior

## Abstract

**Background:**

Guided by the Socialization Model of Child Behavior (SMCB), this cross-sectional study examined direct and indirect associations of parental cognitions and behavior, the home and neighborhood environment, and toddlers’ personal attributes with toddlers’ physical activity and screen time.

**Methods:**

Participants included 193 toddlers (1.6 ± 0.2 years) from the Parents’ Role in Establishing healthy Physical activity and Sedentary behavior habits (PREPS) project. Toddlers’ screen time and personal attributes, physical activity- or screen time-specific parental cognitions and behaviors, and the home and neighborhood environment were measured via parental-report using the PREPS questionnaire. Accelerometry-measured physical activity was available in 123 toddlers. Bayesian estimation in structural equation modeling (SEM) using the Markov Chain Monte Carlo algorithm was performed to test an SMCB hypothesized model. Covariates included toddlers’ age, sex, race/ethnicity, main type of childcare, and family household income.

**Results:**

In the SMCB hypothesized screen time model, higher parental barrier self-efficacy for limiting toddlers’ screen time was associated with higher parental screen time limiting practices (*β* = 0.451), while higher parental negative outcome expectations for limiting toddlers’ screen time was associated with lower parental screen time limiting practices (*β* = − 0.147). In turn, higher parental screen time limiting practices was associated with lower screen time among toddlers (*β* = − 0.179). Parental modeling of higher screen time was associated with higher screen time among toddlers directly (*β* = 0.212) and indirectly through the home environment. Specifically, higher screen time among parents was associated with having at least one electronic device in toddlers’ bedrooms (*β* = 0.146) and, in turn, having electronics in the bedroom, compared to none, was associated with higher screen time among toddlers (*β* = 0.250). Neighborhood safety was not associated with toddlers’ screen time in the SEM analysis. No significant correlations were observed between the SMCB variables and toddlers’ physical activity; thus, no further analyses were performed for physical activity.

**Conclusions:**

Parents and their interactions with the home environment may play an important role in shaping toddlers’ screen time. Findings can inform family-based interventions aiming to minimize toddlers’ screen time. Future research is needed to identify correlates of toddlers’ physical activity.

## Background

Accumulating evidence suggests that physical activity has favorable effects, whereas screen-based sedentary behavior or screen time (e.g., television [TV] viewing) has detrimental effects on the health and well-being of children in the early years (birth to 4 years) [1–3] and school-aged children and youth (5–17 years) [4, 5]. Given that physical activity and screen time patterns in the early years tend to persist into later childhood and adolescence [6, 7], it is imperative that healthy active lifestyles are established early. However, recent prevalence estimates in a representative sample of Canadian children in the early years [8] indicate that 62% meet physical activity recommendations, 24% meet screen recommendations, and collectively 15% meet both recommendations within the new *Canadian 24-Hour Movement Guidelines for the Early Years* [9]. Therefore, identifying the key correlates of health-enhancing behavioral patterns of regular physical activity and minimal screen time during these formative years is of great importance.

It is well established that parents have an overarching influence on children’s physical activity and screen time participation in the early years. Specific parenting practices may facilitate (e.g., parental modeling, parental support) or limit (e.g., TV viewing time rules) children’s behaviors [10–12]. Parents also create a home environment that shapes and reinforces physical activity and screen time. For example, parents have direct control over what types of physical activity equipment or electronics are available in the home [13]. In addition, characteristics of neighborhoods where families live may also assist in enabling or disabling physical activity and screen time [14, 15]. However, to the authors’ knowledge, no study has investigated the interplay between individual, familial, and environmental correlates and their combined associations with physical activity and screen time among children of the early years.

Theories provide structure for understanding the correlates of behavior. However, few behavioral theories or models focus specifically on children’s behavior [16–18]. The Socialization Model of Child Behavior (SMCB) [17, 19] is one such model that incorporates the environment (e.g., neighborhood safety, availability of electronic devices/physical activity equipment at home), parental cognitions (e.g., parental positive/negative outcome expectations for supporting physical activity and limiting screen time) and behaviors (e.g., parental physical activity and screen time), and children’s cognition/personal attributes (e.g., children’s activity temperament) as correlates of children’s behavior (e.g., physical activity, screen time). The model postulates that the correlates of behavior reciprocally interact with each other. The model also proposes direct and indirect associations between correlates and children’s behaviors. Specifically, parental behaviors, children’s cognitions or personal attributes, and the environment are thought to be directly associated with children’s behaviors. In addition, parental cognitions are thought to be associated with children’s behaviors indirectly through parental behaviors and the environment. No studies have used the full SMCB to guide the examination of correlates of children’s physical activity and screen time.

Though the SMCB has not specifically been tested before, a number of reviews have examined the individual correlates of physical activity and/or screen time in the early year age group [12, 20–23]. However, the majority of evidence is focused on preschool-aged children (4–5 years) with little attention given to younger children [24–26]. Toddlerhood (12–35 months) is an optimal period for targeted interventions as most children become ambulatory and are being introduced to regular screen time during the ages of 2 to 3 years [22, 27]. Given the rapid growth and development during early childhood, intervention targets needed for toddlers may be different from those of preschoolers, and therefore interventions may need to be adapted to match the appropriate developmental level.

**Fig. 1:**
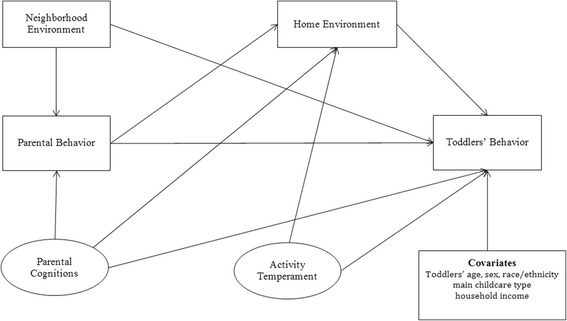
A hypothesized model explaining the associations between parental cognitions, parental behavior, and the home and neighborhood environment on toddlers’ screen time. Note: Ovals represent latent constructs and rectangles represent measured variables

The purpose of this study was to examine direct and indirect associations of the home and neighborhood environment, parental cognitions and behavior, and toddlers’ personal attributes with toddlers’ physical activity and screen time (see Fig. 1). Specifically, it was hypothesized that (1) the neighborhood environment would be associated with toddlers’ physical activity and screen time directly and indirectly through parental behavior and the home environment; (2) parental cognitions would be associated with toddlers’ physical activity and screen time indirectly through parental behavior and the home environment; and (3) activity temperament would be associated with toddlers’ physical activity and screen time directly and indirectly through the home environment. In addition, it was hypothesized that parental cognitions and parental behavior would have the strongest direct and indirect associations with toddlers’ physical activity and screen time.

## Methods

### Participants

Data was sourced from the baseline time point (October, 2014 to September, 2015) of the *Parents’ Role in Establishing healthy Physical activity and Sedentary behavior habits* (PREPS) project. Participants were recruited from four large local health centers in socioeconomically diverse neighborhoods in Edmonton, Canada during children’s 18-month immunization appointments. Families were eligible to participate in the PREPS project if: (1) toddlers were walking, and (2) a parent was able to speak and read English. Of the 491 eligible families, a total of 257 agreed to participate in the study (participation rate: 52%). The remaining 234 declined to participate due to the following reasons: busy schedules/lack of time/fatigue (*n* = 74), no interest (*n* = 64), parental perception that their child would not wear the accelerometer belt (*n* = 60), travel/illness/moving away (*n* = 20), or a parent not being present at the time of data collection (*n* = 16).

### Procedures

During the 15-min waiting period required after children’s immunizations, research staff asked eligible and participating families to complete a consent form and the PREPS questionnaire. Missing data were minimized by checking questionnaires for completeness and following up with families when needed. Participating families were also provided an accelerometer at the immunization appointment for their toddler to wear on the right hip for seven consecutive days, except for nighttime sleep and water-based activities (e.g., swimming, bathing). A pre-paid courier return envelope and written instructions were also provided. A mid-week reminder was sent to participating parents about the continuous wear of their toddler’s accelerometer. Informed written consent was provided by each participating parent, and ethics approval was granted by the University of Alberta Human Research Ethics Board. Detailed information about the PREPS project is described elsewhere [19, 28].

### Measures

The PREPS questionnaire captured demographic information as well as physical activity and screen time information for each construct of the SMCB. The questionnaire was informed by a pilot study and through review of the literature [19]. Detailed descriptions of the variables and items of the PREPS questionnaire as well as the corresponding psychometric properties have been published previously [19]. Some variables were specific to toddlers’ physical activity or screen time and other variables applied to both.

#### Children’s behavior

Toddlers’ physical activity and screen time were the variables assessed with the children’s behavior construct of the SMCB. Toddlers’ physical activity was objectively measured in 15-s epochs using waist-worn ActiGraph wGT3X-BT (ActiGraph Corp, Pensacola, FL, USA) accelerometers. Non-wear time was defined as ≥80 consecutive 15-s intervals of zero counts (equivalent to ≥20 min of consecutive zero counts). Based on previous reliability estimates, toddlers with ≥4 days of valid data with ≥1440 total 15-s intervals (i.e., ≥ 6 h of wear time) were included in the analysis [24, 29]. Daytime naps were assumed to be included in non-wear time. Light-intensity physical activity (LPA) was defined as 25–420 counts/15-s epoch and moderate- to vigorous-intensity physical activity (MVPA) was defined as > 420 counts/15-s epoch [25, 30]. Minutes per day of LPA and MVPA were calculated by dividing the number of 15-s intervals by four and then dividing by the total number of valid days. Minutes per day of total physical activity (TPA) was then calculated by summing minutes per day of LPA and MVPA. Wear time standardized variables were used in all analyses by obtaining the residual values calculated from regressing the physical activity variables on wear time [31]. Previous research has reported strong criterion validity of the accelerometer against direct observation for measuring physical activity in toddlers (*r* = 0.66–0.82) [32, 33].

Toddlers’ screen time was subjectively measured via parental-report using four items adopted from previous studies [34, 35] that were modified from the Canadian Health Measures Survey [36]. Specifically, parents were asked to report on the average hours and minutes per day on weekdays and weekend days for: (1) TV, videos, or DVDs on a TV, computer, or portable device, and (2) video/computer games on electronic devices (e.g., a learning laptop, leapfrog leapster, computer, laptop, tablet, cell phone, the internet, Playstation, or XBOX). Total TV viewing and video/computer game use were computed using weighted averages for weekday and weekend responses (i.e., [weekday*5 + weekend*2]/7). Weighted minutes per day of each variable were then summed to generate total screen time. Good one-week test-retest reliability was shown for the screen time questions (Intraclass correlation [ICC] = 0.82) [19].

#### Children’s personal attributes

Toddlers’ activity temperament was the variable assessed within the children’s personal attributes construct of the SMCB for physical activity and screen time. Activity temperament included 12 items from the short form of Early Childhood Behavior Questionnaire [37]. Parents were asked to report the frequency of specific behaviors their toddler engaged in (e.g., “sit quietly”, “seem full of energy even in the evening”, “toss about in bed”, “run through the house”) within six different contexts (i.e., while bathing, when participating in daily activities, during sleep, when playing outdoor with other children, while being dressed or undressed, while playing indoors) on a 7-point scale ranging from “never” to “always”. After reverse coding four items, responses from the 12 items were averaged; higher average estimates represented a higher “predilection for movement” [38]. Good one-week test-retest reliability (ICC = 0.78) and good internal consistency (α = 0.79–0.84) at two time points were shown for the activity temperament questions [19].

#### Parental cognitions

Barrier self-efficacy (i.e., one’s belief about one’s ability to complete a task while overcoming difficulties [39]) and outcome expectations (i.e., a belief about the likelihood of the behavior leading to a specific outcome [40]) for supporting toddlers’ physical activity and limiting screen time were the variables assessed within the parental cognitions construct of the SMCB. Barrier self-efficacy for supporting toddlers’ physical activity included 7 items primarily based on a previous study [41]. All items were rated on an 8-point scale ranging from “not confident” to “completely confident”. Outcome expectations for supporting toddlers’ physical activity included 8 items (5 items reflecting positive outcome expectations and 3 items reflecting negative outcome expectations) primarily based on a previous study [41]. All items were rated on an 8-point scale ranging from “no chance” to “certain to happen”. Responses for barrier self-efficacy, positive outcome expectations, and negative outcome expectations were averaged within the respective scales. Higher average estimates represented higher confidence for overcoming barriers related to supporting toddlers’ physical activity and higher positive or negative perceived consequences for supporting toddlers’ physical activity. Moderate one-week test-retest reliability and moderate to good internal consistency at two time points were shown for the barrier self-efficacy (ICC = 0.62; α = 0.84–0.86), positive outcome expectations (ICC = 0.62; α = 0.90–0.95), and negative outcome expectations (ICC = 0.55; α = 0.61–0.68) for supporting physical activity questions [19].

Barrier self-efficacy for limiting screen time included 3 items primarily based on previous studies [35, 41, 42]. All items were rated on an 8-point scale ranging from “not confident” to “completely confident”. Outcome expectations for limiting screen time included 7 items (3 items reflecting positive outcome expectations and 4 items reflecting negative outcome expectations) primarily based on a previous study [43]. All items were rated on an 8-point scale ranging from “no chance” to “certain to happen”. Responses for barrier self-efficacy, positive outcome expectations, and negative outcome expectations were each averaged. Higher average estimates represented higher confidence for overcoming barriers to limiting toddlers’ screen time and higher positive or negative perceived consequences of limiting toddlers’ screen time, respectively. Moderate one-week test-retest reliability and good internal consistency at two time points were shown for the barrier self-efficacy (ICC = 0.64; α = 0.86), positive outcome expectations (ICC = 0.62; α = 0.92–0.93), and negative outcome expectations (ICC = 0.64; α = 0.74–0.77) for limiting screen time questions [19].

#### Parental behavior

Modeling of physical activity and support for physical activity, and modeling of screen time and limits on screen time were the variables assessed within the parental behavior construct of the SMCB. Modeling of physical activity included 6 items on physical activity adopted from the short form of the International Physical Activity Questionnaire [44]. Parents were asked to report the duration and frequency of vigorous intensity physical activity (VPA), moderate intensity physical activity (MPA), and walking during the last 7 days. Time spent in VPA, MPA, and walking activity were weighted by the energy expended for each activity to produce MET-minutes of PA values per week (i.e., 8.0*VPA minutes*VPA days + 4.0*MPA minutes*MPA days + 3.3*walking minutes*walking days). Support for physical activity included 4 items based on previous studies [19, 45, 46]. All items were rated on an 8-point scale ranging from “never” to “daily”. Higher estimates represented more frequent parental support for physical activity. Moderate to good one-week test-retest reliability were shown for the physical activity modeling (ICC = 0.67), and physical activity support questions (ICC = 0.59), and good internal consistency at two time points were also shown for the physical activity support question (α = 0.74–0.77) [19].

Modeling of screen time included 3 items adopted from a national survey in Canada [36]. Parents were asked to report on the duration of their weekly screen viewing in the past 3 months by hours and minutes. Total TV viewing, computer use, and video game use were averaged and then summed to generate parents’ total screen time per day (i.e., [TV viewing hours*60 + TV viewing minutes]/7 + [computer use hours*60 + computer use minutes]/7 + [video game use hours*60 + video game use minutes]/7). Parents’ screen time data were presented as minutes per day by dividing the values by seven to be consistent with the toddlers’ data. Limits on screen time included a single item primarily based on a previous study [47]. This single item was rated on an 8-point scale ranging from “never” to “daily”. Higher estimates represented more frequent limiting of toddlers’ screen time to zero minutes per day. Moderate to good one-week test-retest reliability were shown for the screen time modeling (ICC = 0.76) and limits questions (kappa = 0.57) [19].

#### Home environment

Yard space and the availability of physical activity equipment at home were the variables assessed within the home environment construct of the SMCB for physical activity. The yard space variable included a single item and the availability of physical activity equipment at home included 10 items based primarily on a previous study [47]. Parents were asked to report on how big their yard is on a 6-point scale ranging from “no yard” to “a large yard (1/4 acre block or larger)”. Availability of physical activity equipment was assessed by asking parents to report on types of toys and equipment available at home for their toddler to engage in physical activity (i.e., balls, basketball hoop, bats/racquets/golf clubs, climbing equipment, gardening tolls, play house, pool or beach toys, tricycle/bicycle, and other). Response options included “yes” and “no”. The total number of physical activity equipment available at home was generated by aggregating these items. Higher estimates represented having more physical activity equipment available in the home. Almost perfect and good one-week test-retest reliability were shown for the yard space (kappa = 0.96) and availability of physical activity equipment questions (ICC = 0.75), respectively [19].

Availability of electronic devices at home and the presence of electronic devices in the toddlers’ bedroom were the variables assessed within the home environment construct of the SMCB. Availability of electronic devices at home included 7 items and the presence of electronic devices in the bedroom included 3 items primarily based on a previous study [47]. Availability of electronic devices was assessed by asking parents to report on the number of electronic devices they have in their house (i.e., TV, video/DVD/Blu-ray payer, desktop computer, laptop, tablet computer, cell phone, and video game console). The total number of electronic devices available in the home was generated by aggregating available items. Higher estimates represented having more screen time devices available in the home. Presence of electronic devices in the bedroom were also assessed by asking parents to indicate if their child had TV/ portable DVD player, computer (e.g., learning laptop, laptop, netbook, iPad, and cellphone) and video game console in their bedroom with response options, “yes” or “no”. A dichotomous variable was generated with “1” having ≥ one electronic device and “0” as having none in the bedroom. Good and almost perfect one-week test-retest reliability were shown for the availability of electronic equipment in the home (ICC = 0.88), and presence of electronic equipment in the bedroom questions (kappa = 0.95), respectively [19].

#### Neighborhood environment

Suitability of playgrounds (physical activity) and neighborhood safety (physical activity and screen time) were the variables assessed within the neighborhood environment construct of the SMCB. Suitability of playgrounds included 3 items and neighborhood safety included a single item adopted from a previous study [47]. Suitability of playgrounds was assessed by asking parents to report on the playgrounds in their local neighborhood (i.e., number of playgrounds, having suitable equipment, free from litter, graffiti, vandalism, and dog droppings) on a 5-point scale ranging from “strongly disagree” to “strongly agree”. Higher estimates represented having more suitable playgrounds. Neighborhood safety was measured by asking parents to report on their perceived safety for their child and themselves to walk/cycle/play during daytime on a 4-point scale ranging from “strongly disagree” to “strongly agree”. Higher estimates represented having a safer neighborhood. Substantial and moderate one-week test-retest reliability were shown for the neighborhood safety (kappa = 0.63) and suitability of playgrounds (ICC = 0.58) questions, respectively [19]. In addition, good internal consistency at two time points were shown for the suitability of playgrounds question (α = 0.80–0.84) [19].

#### Covariates

Covariates included toddlers’ age, sex, race/ethnicity, main type of childcare, and household income based on a previous study from the PREPS project [28]. Parents were asked to report on their toddlers’ birthdate, sex (male or female), race/ethnicity (i.e., Aboriginal/First Nation, African-Canadian, Arabic, Asian/Pacific Islander, European Canadian/Caucasian, Hispanic/Latino/Latina, or Other), hours per week spent in care other than parents (i.e., daycare center, home daycare, another adult in your home, another adult outside your home, other), and gross household income over the past 12 months (quartiles ranging from <$25,000 to > $100,000, or ‘do not know’). Consistent with previous studies involving the current sample [28, 48], toddlers’ race/ethnicity was categorized into two groups (i.e., European-Canadian/Caucasian and Non-European-Canadian/Non-Caucasian), household income was categorized into three groups (i.e., ≤ $50,000, $50,001–$100,000, and > $100,000), and the main type of childcare was categorized into four groups (i.e., parental care, childcare center, home daycare, and other).

### Statistical analysis

The data were evaluated for normality, linearity, homoscedasticity, multicollinearity, and for potential outliners. Two toddlers and five parents with extremely high screen time values (≥ ±3 standard deviations [SD]) were truncated below ±3 SD. In addition, 22 parents who exceeded 180 min/day of walking, MPA, or VPA variables were truncated to be equal to 180 min/day according to the IPAQ scoring protocol (www.ipaq.ki.se). All continuous variables were within an acceptable range of kurtosis and skewness (±2) [49–51], thus, no transformations were made. Means and standard deviations (M ± SD) for continuous variables and frequencies (%) for categorical variables were calculated to describe participant characteristics. Spearman’s rank correlations were performed to examine correlations between the SMCB variables. Statistical significance was set at *p* < 0.05. To create the most parsimonious models, physical activity variables not significantly correlated with toddlers’ physical activity and screen time variables not significantly correlated with toddlers’ screen time were removed from the hypothesized model before the main analysis. The strength of a correlation coefficient was defined as weak if coefficients were below 0.3, moderate if coefficients ranged from 0.3 to 0.5, and strong if coefficients were greater than 0.5 [52].

To operationalize the SMCB [17] for structural equation modeling (SEM), an adapted version was developed in accordance with the previous literature pertaining to the correlates of physical activity and screen time among children in the early years [12, 20–23] and statistical principles of SEM [53] (see Fig. 1). The adapted model included three main changes from the original SMCB. First, the directionality of associations between constructs was specified. Second, a direct path between parental cognitions and children’s behavior was added so the indirect effect of parental cognitions on children’s behavior and the environment could be estimated [53]. Finally, the environment was separated into the home and neighborhood environment because the directionality of associations was thought to be different between these two environment settings [54–56].

Since a binary outcome was part of our hypothesized model (i.e., having versus not having electronics in the bedroom), the Markov Chain Monte Carlo algorithm (MCMC) methods were employed to obtain Bayesian estimation in SEM. Unlike frequentist analysis (e.g., maximum likelihood), Bayesian analysis provides parameter estimates from background knowledge (i.e., prior) to inform new data (i.e., likelihood), and MCMC sampling methods achieve these estimates from the mean of the posterior distribution [57, 58]. Therefore, Bayesian and frequentist methods differ in their definition of probability. With the frequentist approach, probability is determined based on one true regression coefficient that is fixed but unknown. However, in Bayesian theorem, probability is a degree of belief from our a priori knowledge that a relationship exists between variables. The likelihood of data is then used to weigh the prior, which yields the posterior distribution in Bayesian theorem [58]. The degree of beliefs in the Bayesian approach can be updated when we have further information (i.e., posterior distribution), while probabilities are estimated directly from samples with confidence intervals in the frequentist approach [58].

In this study, the relationships between variables are largely unknown, therefore we used uninformative (diffuse) prior. This method is thought to have minimum impact on the model estimates [59]. The hypothesized model (Fig. 1) was fitted after controlling for toddlers’ age, sex, race/ethnicity, main childcare type, and household income. Standardized estimates (i.e., a single measure of the middle of the posterior distribution) and 95% credible intervals (95% CI) for posterior estimates were reported. The Bayesian credible interval is interpreted as a probability statement about the parameter itself. For example, Prob (*a* ≤ *θ* ≤ *b*) = 0.95) indicates that one is 95% sure that the true value of *θ* lies between *a* and *b* [58]. Stable parameter estimates were determined when the convergence statistic was less than 1.002 and when each Bayesian SE had a value lower than 0.05 [60]. Model fit was checked by computing Bayesian posterior predictive *p* values. The model was determined as well-fitted when posterior predictive *p* value was close to 0.50 [58]. In Bayesian inference, plausible reasoning, instead of frequentist’s significant testing, is employed. Plausible reasoning attempts to validate or invalidate hypotheses using uncertain information and can be used to reason about the truth of single hypothesis (H or ¬H) or choose from a number of competing hypotheses (H1) [61]. In this study, plausibility refers to the degree to which a statement can be believed and can be represented by likelihood or probability [61]. Specifically, a plausible association refers to the probability that a regression coefficient resides between the upper and lower limits of 95% CIs. Prior to Bayesian SEM, latent variables were created for variables with multiple items. IBM SPSS Statistics 20 and IBM SPSS AMOS 20 (IBM Corp, NY) were used to perform the statistical analyses.

## Results

A total of 203 out of the 257 participants had complete data on the key SMCB variables from the PREPS questionnaire. As for demographic variables, 12 participants did not respond or responded “do not know” to the household income question and one participant did not indicate race/ethnicity, leaving a total sample of 193 toddlers for the analyses involving screen time. Between the samples of included (*n* = 193) and excluded (n = ranged from 44 to 64 due to missing cases), no significant differences were observed in toddlers’ age (1.6 ± 0.2 vs. 1.6 ± 0.4), sex (boys: 49.2% vs. 47.8%), race/ethnicity (Caucasian/European-Canadian descent: 53.9% vs. 55.6%), household income (15.5 vs. 27.5% in ≤ $50,000; 38.3% vs. 37.3% in $50,000–$100,000; 46.1% vs. 35.3% in > $100,000), or the main type of childcare (35.8% vs. 23.1% in parental care; 18.7% vs. 15.4% in childcare center; 14.0% vs. 13.8% in home daycare; 31.6% vs. 47.7% in other).

A total of 123 out of the 193 participants provided complete accelerometry data and were included in the analyses involving physical activity. Between samples of included (*n* = 123) and excluded (*n* = 70) participants, no significant differences were observed in toddlers’ age (1.6 ± 0.2 vs. 1.6 ± 0.3), sex (boys: 53.7% vs. 45.7%), race/ethnicity (Caucasian/European-Canadian descent: 58.5% vs. 45.7%), household income (12.2 vs. 21.4% in ≤ $50,000; 39.8% vs. 35.7% in $50,000–$100,000; 48.0% vs. 42.9% in > $100,000), or the main type of childcare (36.6% vs. 34.3% in parental care; 18.7% vs. 18.6% in childcare center; 14.6% vs. 12.9% in home daycare; 30.1% vs. 34.3% in other). A detailed description of participant characteristics is presented in Table 1. The average time spent in front of a screen was 101.8 ± 108.8 min/day among toddlers. Toddlers spent an average of 298.0 ± 39.9 min/day in TPA, with 58.7 ± 18.7 min in MVPA.

**Table 1:** Demographic and Socialization Model of Child Behaviorcharacteristics among toddlers and their parents

	*n* = 193
Demographic characteristics	
Toddlers’ age	1.6 ± 0.2
Toddlers’ sex	
Boys	50.8 (98)
Girls	49.2 (95)
Toddlers’ race/ethnicity	
European-Canadian/Caucasian	53.9 (104)
Other^a^	46.1 (89)
Household income	
< $50,000	15.5 (30)
$50,001 to $100,000	38.3 (74)
≥ $100,000	46.1 (89)
Main type of childcare	
Parental	35.8 (69)
Other^b^	64.2 (124)
SMCB characteristics	
*Activity temperament* (0–7)	5.5 ± 0.8
*Screen time*	
TV (min/day)	92.7 ± 147.5
VG (min/day)	15.5 ± 38.7
Total screen time (TV + VG; min/day)	101.8 ± 108.8
*Physical activity* (*n* = 123)	
LPA (min/day)	239.1 ± 27.5
MVPA (min/day)	58.7 ± 18.7
TPA (min/day)	298.0 ± 39.9
*Screen time: Parental behavior*	
Screen time modeling	
TV (min/day)	80.8 ± 88.5
Computer (min/day)	77.4 ± 107.6
VG (min/day)	1.8 ± 7.8
Total screen time (TV + Computer + VG; min/day)	147.5 ± 114.9
Limit on toddlers’ screen time (0–7)	2.8 ± 2.5
*Screen time: Parental cognition*	
Positive outcome expectations (0–7)	4.6 ± 2.2
Negative outcome expectations (0–7)	2.5 ± 1.6
Barrier self-efficacy (0–7)	4.6 ± 1.9
*Physical activity: Parental behavior* (n = 123)	
Physical activity modeling	
Total physical activity (MET·min·week^−1^)	3198.8 ± 2902.7
Physical activity support (0–7)	4.9 ± 1.4
*Physical activity: Parental cognition* (n = 123)	
Positive outcome expectations (0–7)	6.5 ± 0.8
Negative outcome expectations (0–7)	4.1 ± 1.6
Barrier self-efficacy (0–7)	4.6 ± 1.3
Home environment	
*Screen time*	
Presence of at least one electronic equipment in bedroom (%)^c^	12.4 (24)
Availability of electronic equipment (Total number)^§^	9.1 ± 3.1
*Physical activity* (*n* = 145)	
Availability of PA equipment (total number)^d^	14.6 ± 1.8
Yard space (1–5)	3.5 ± 1.0
Suitability of playgrounds (1–5)	3.9 ± 0.8
Neighborhood environment	
Neighborhood safety (1–4)	3.3 ± 0.7

Data are presented as mean ± standard deviation for continuous/ordinal variables and percentages (n) for dichotomous variables

SMCB = Socialization Model of Child Behavior

^a^Non-European-Canadian/Non-Caucasian included aboriginal/First Nation, African-Canadian, Arabic, Asian/Pacific Islander, Hispanic/Latino/Latina, and others (self-expressed)

^b^Other included childcare center, home daycare, and another adult (e.g., friend, relative, nanny, baby sitter) in and outside home

^c^Electronic equipment included TV, video/DVD/Blu-ray player, desktop computer, laptop, tablet computer, cell phone, and video game console

^d^PA equipment included balls, basketball hoop, bats/racquets/golf clubs, climbing equipment, gardening tools, play house, pool or beach toys, tricycle/bicycle, and other (self-described)

Correlations between SMCB screen time variables are summarized in Table 2. Variables that were significantly negatively correlated with toddlers’ screen time included parental limits on toddlers’ screen time (*r* = − 0.407), barrier self-efficacy for limiting screen time (*r* = − 0.432), and neighborhood safety (*r* = − 0.310). Variables that were significantly positively correlated with toddlers’ screen time included parental modeling of screen time (*r* = 0.302), negative outcome expectations for limiting screen time (*r* = 0.414), and having at least one electronic device in the bedroom (*r* = 0.312). The strength of these correlations was moderate. Three variables not significantly correlated with toddlers’ screen time (i.e., activity temperament [*r* = 0.118], positive outcome expectations for limiting toddlers’ screen time [*r* = − 0.073], and total number of electronic devices at home [*r* = − 0.061]) were removed from the hypothesized model and excluded from further analysis. No significant correlations were observed between the SMCB physical activity variables and MVPA or TPA (Table 3) or between SMCB physical activity variables and LPA (data not shown). Therefore, SEM for the physical activity outcome was not performed.

**Table 2:** Spearman rank correlations (one-tailed) between measures of the home and neighborhood environments, parental cognitions and modeling, and toddlers’ temperament and screen time (*n* = 193)

	1	2	3	4	5	6	7	8	9
Toddler Behavior									
1. Screen time									
Toddler Cognition/Personal Attributes									
2. Temperament	.118								
Parent Cognition/Behavior									
3. Screen time	.302**	.072							
4. Limit^a^	−.407**	−.120*	−.198**						
5. Positive OE^a^	−.073	−.001	−.045	.194**					
6. Negative OE^a^	.414**	−.102	.145*	−.267**	.021				
7. Barrier SE^a^	−.432**	.000	−.180**	.454**	.165*	−.431**			
Home environment									
8. Bedroom^b^	.312**	.152*	.134*	−.039	−.015	.155*	−.186**		
9. Availability^b^	−.061	−.083*	.104	.038	−.145*	.035	−.075	.052	
Neighborhood environment									
10. Safety	−.310**	−.006	−.140*	.047	.120*	−.188**	.176**	−.262**	.069

^a^Limit: Parental limit on screen time; *OE* Outcome expectations, *SE* Self-efficacy

^b^Bedroom: Presence of electronic equipment in bedroom; Availability: Total number of electronic equipment at home

*p < .05; **p < .01

**Table 3:** Spearman rank correlations (one-tailed) between measures of the home and neighborhood environments, parental cognition and physical activity, and toddlers’ temperament, and MVPA (non-shaded)\TPA (shaded) (*n* = 123)

	1	2	3	4	5	6	7	8	9	10	11
Toddler Behavior											
1. MVPA\TPA		.036	.040	−.057	−.088	−.113	−.097	.006	.043	.042	.060
Toddler Cognition/Personal Attributes											
2. Temperament	−.043		.052	−.062	.055	−.019	−.097	.115	−.084	.009	−.034
Parent Cognition/Behavior											
3. MET·min·week^−1^	.070	.052		.228**	.128	−.047	.110	−.162*	.082	−.004	.055
4. PA support	−.131	−.062	.228**		.351***	−.065	.355***	−.248**	.290**	.201*	.211*
5. Positive OE^a^	−.050	.055	.128	.351***		.111	.333***	−.179*	.173*	.080	.110
6. Negative OE^a^	−.065	−.019	−.047	−.065	.111		.074	−.020	−.142	−.198*	−.199*
7. Barrier SE^a^	−.130	−.097	.110	.355***	.333***	.074		−.265**	.026	.172*	.088
Home environment											
8. Availability^b^	.017	.115	−.162*	−.248**	−.179*	−.020	−.265**		−.250**	−.081	−.053
9. Yard Space	−.004	−.084	.082	.290**	.173*	−.142	.026	−.250**		.183*	.171*
Neighborhood environment											
10. Suitability^b^	.068	.009	−.004	.201*	.080	−.198*	.172*	−.081	.183*		.380***
11. Safety^‡^	−.036	−.034	.055	.211*	.110	−.199*	.088	−.053	.171*	.380***	

^a^*OE* Outcome expectations, *SE* Self-efficacy

^b^Availability: Total number of PA equipment at home; Suitability: Suitability of playgrounds; Safety: Neighborhood safety

*p < .05; **p < .01; ***p < .001

Based on the results of the correlation analyses, the following variables were included in the SEM for screen time: negative outcome expectations and barrier self-efficacy of screen time limits, limits on screen time, parental modeling of screen time, presence of electronic equipment in bedroom, and neighborhood safety (Fig. 1). Two variables existed for parental cognitions (i.e., negative outcome expectations, barrier self-efficacy), and parental behavior (i.e., parental limits on toddlers’ screen time, parental modeling of screen time) constructs. The SMCB hypothesized model met the assumptions for stable parameter estimates with a convergence statistic of ≤1.001. Additionally, Bayesian standard errors for each parameter estimate was lower than 0.03. The SMCB hypothesized model was also shown to be well-fitted (Posterior predictive *p* = 0.48) (see Appendix A).

**Fig. 2:**
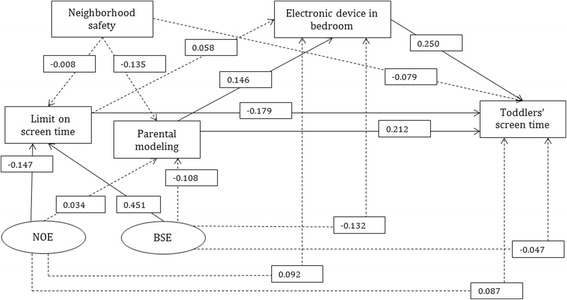
The SMCB hypothesized screen time model (*n* = 193). Note: All solid line parameters are within 95% credible intervals; dashed line parameters are not within 95% credible intervals. Ovals represent latent constructs and rectangles represent measured variables. Only standardized beta coefficients are presented and the error terms, covariates, 95% credible intervals and measurements are not presented for clarity. Analysis was adjusted for toddler’s age, sex, race/ethnicity, main childcare type and household income. NOE: Negative outcome expectations; BSE: Barrier self-efficacy

The SMCB hypothesized screen time model is shown in Fig. 2. No plausible direct associations were observed between parental cognitions and toddlers’ screen time. However, plausible indirect associations were observed between parental cognitions and toddlers’ screen time, through parental behaviors. Specifically, higher negative outcome expectations for limiting toddlers’ screen time was associated with lower parental limits on toddlers’ screen time (*β* = − 0.147, 95% CI = − 0.169, − 0.125), and in turn, lower parental limits on toddlers’ screen time was associated with higher screen time among toddlers (*β* = − 0.179, 95% CI = − 0.309, − 0.052) Similarly, higher barrier self-efficacy for limiting toddlers’ screen time was associated with higher parental limits on toddlers’ screen time (*β* = 0.451, 95% CI = 0.315, 0.572), and in turn, higher parental limits on toddlers’ screen time was associated with lower screen time among toddlers (*β* = − 0.179, 95% CI = − 0.309, − 0.052).

Parental modeling did not mediate the relationship between parental cognitions and toddlers’ screen time. However, higher parental modeling of screen time was directly associated with higher screen time among toddlers (*β* = 0.212, 95% CI = 0.099, 0.325). In addition, higher parental modeling of screen time was associated with having at least one electronic device in the bedroom (*β* = 0.146, 95% CI = 0.010, 0.277) and, in turn, having at least one electronic device in the bedroom, compared to having no electronic device in the bedroom, was associated with higher screen time among toddlers (*β* = 0.250, 95% CI = 0.130, 0.365). No plausible direct and indirect associations were observed between neighborhood safety and toddlers’ screen time.

## Discussion

To the authors’ knowledge, this study was the first to use the SMCB to guide the comprehensive examination of the key correlates of physical activity and screen time in the early years age group. The interplay of important correlates associated with toddlers’ screen time was identified. As hypothesized, and in line with the SMCB model, parental cognitions (negative outcome expectations, barrier self-efficacy for limiting screen time) and behaviors (parental limits on screen time, parental modeling of screen time), and the home environment (presence of electronic devices in the bedroom) were independently and simultaneously associated with toddlers’ screen time. Specifically, parental cognitions were indirectly associated with toddlers’ screen time through parental screen time limits. Parental modeling of screen time was directly associated with toddlers’ screen time and indirectly through having at least one device in the bedroom. However, neighborhood safety, which was significantly correlated with toddlers’ screen time was not associated with toddlers’ screen time in the SEM analysis. Additionally, no correlates of physical activity, in terms of TPA, LPA, or MVPA were identified.

The finding in the present study that parental cognitive factors were significantly correlated with toddlers’ screen time is consistent with two previous studies [34, 62]. Specifically, higher parental self-efficacy to limit screen time was associated with lower screen time among Canadian children aged 0 to 5 years [34] and Australian children aged 1 to 5 years [62]. However, it is counterintuitive to hypothesize that parental cognitions will have a direct effect on children’s behavior because cognitions are intrinsic to individuals and one’s cognitions can only be directly translated into the behavior of their own and not to the behavior of others [63]. As Taylor and colleagues [17] stipulated in the SMCB model, the association between parental cognitions and children’s behavior is likely due to one or more parental behavior variables that mediate the association. Through the use of SEM, the present study provided evidence for this indirect association. Specifically, negative outcome expectations and barrier self-efficacy for limiting screen time were not directly associated with toddlers’ screen time; rather, they were indirectly associated with toddler’ screen time through parental limits on toddler’s screen time. Unlike negative outcome expectations and barrier self-efficacy, positive outcome expectations did not appear to be an important correlate of toddler’s screen time. This may be explained by the fact that zero screen time is often seen as unrealistic and impractical by parents [64, 65], and the negative outcomes of limiting screen time assessed in this study represented more practical day to day challenges (e.g., less time for parents to do other things, disruption of family practices and routines, unhappy child) than the potential positive outcomes assessed (e.g., improvements to child health, mood/behavior, social skills). Given the dearth of evidence in this area, future research is needed to confirm and build on these findings.

Parental cognitive factors were not associated with parental modeling of screen time; however, parental modeling of screen time was directly associated with toddlers’ screen time. Combined, the findings from this study add to the growing evidence that parental behavior, including limits and modeling, is important in shaping screen time behavior during early childhood [12, 22]. Though not examined in this study, parental limits and role modeling with respect to screen time may also interact with one another, and simultaneously influence toddlers’ screen time. For instance, De Decker and colleagues [66] found that parental rules about TV viewing time mediate the relationship between parents’ and preschoolers’ TV viewing time in Australian and Belgian samples. Also, in line with the SMCB model [17], findings of the present study suggest that parental screen time modeling is not only directly associated with toddlers’ screen time but also indirectly through the home environment. This finding builds upon previous literature [12, 21, 66–69] that has only considered the direct effect between parental modeling and toddler’s screen time behavior. It is possible that parents who spend longer hours in front of a screen may create the home environment that is conducive to screen time for their toddlers. Therefore, creating healthy home environments may not only have implications for toddlers but for the whole family unit.

The roles electronic devices play in young families’ lives are an important area of future inquiry to inform the creation of healthy home environments. Findings suggest the location of the electronic devices (i.e., bedroom) is a more important correlate of toddlers’ screen time than the total number of devices in the home. Interestingly, having an electronic device in the bedroom is a consistent correlate of screen time across early years and school-aged groups [69–73]. Therefore, creating healthy home environments with screen-free bedrooms from an early age may positively impact screen time throughout childhood. Common reasons for having a TV in the bedroom of a child in the early years, including helping children fall asleep, keeping children occupied, and rewarding children for good behavior [74], should be taken into consideration when encouraging families to create healthy home environments. In addition, given the portability of electronic devices (e.g., tablets, laptops, cellphones) in today’s homes, placing parameters on where devices are permitted in the home should also be considered in future efforts to minimize screen exposure among toddlers.

In contrast to the home environment, the neighborhood environment was not associated with toddlers’ screen time in the SEM analysis. Unlike the home environment, parents do not have direct control over the environment of their neighborhood apart from the potential selection of what neighborhood to live in. Thus, neighborhood safety was included as an exogenous variable in which its value is not dependent on other variables in the model. More empirical evidence exists with older children and adolescents that neighborhood safety is associated with behaviors including screen time [20, 42, 75–77]. Conversely, only one study showed the association between neighborhood safety and screen time among 3-year-olds [78], and no corresponding evidence is available in toddlers. While evidence is lacking, it is possible that the immediate environment (i.e., home) is more important for younger children, whereas the distal environment (i.e., neighborhood) is more important for older age groups [16, 75, 79]. For instance, in the present study while a moderate negative correlation was observed between neighborhood safety and toddlers’ screen time, no association was observed when neighborhood safety was fitted in the SMCB hypothesized model with other important parental and home environment correlates. This suggests that the relative predictive importance of neighborhood safety is not as important as other independent variables included in the model simultaneously [54]. Further research is needed to identify at what age the neighborhood environment becomes increasingly important for screen time.

This study was the first to demonstrate the dynamic and complex roles of parental cognitions, parental behaviors, and the home environment in determining toddlers’ screen time patterns. Findings can help to inform targets for future interventions aiming to establish healthy screen time behaviors among toddlers. For example, identifying types of support that parents need in limiting toddler’s screen time, and subsequently providing appropriate, feasible, and sustainable resources and strategies is warranted. Also, providing opportunities to cultivate confidence in encouraging alternative behaviors (e.g., reading books, dancing around, playing indoor games) may increase parental barrier self-efficacy and decrease negative outcome expectations for limiting toddlers’ screen time. Given that maternal self-efficacy for limiting TV viewing has shown to track over time [80], targeting parents early before the onset of children’s screen time exposure may be of importance. Similarly, early intervention in regard to the home environment also appears important, given that parents with higher screen time were more likely to have a toddler who had an electronic device in their bedroom, an important predictor of screen time in younger and older children [69, 72]. In addition, interventions targeting parental behavioral change should be specific to the parental behavior that the intervention is trying to modify. For example, parental screen time limiting practices should be targeted along with changing parental cognitions; whereas, parental modeling should be targeted along with changing the home environment. It is also important to note that interventions to minimize toddlers’ screen time may need to target both parents’ and toddlers’ screen time to yield successful outcomes. However, mothers were the primary participants in this study (87.6%). Qualitative evidence suggests that limiting screen time is challenging for a new parent when their spouse likes watching TV or playing video games [81]. Therefore, future research is required to confirm these findings in other main caregivers in the home (i.e., father, spouse, or common-law partner) to inform family-based interventions that include the entire family unit where applicable.

In contrast to toddlers’ screen time, findings of the present study with regard to toddlers’ physical activity were not supported by the SMCB model. It is possible that other important correlates of toddlers’ physical activity may have been missed. Previous studies examining individual correlates of physical activity in the toddler age group have observed some significant associations with correlates not examined in the present study. For example, more maternal-child interactions [82] and lower parental hostility were found to be associated with higher physical activity among toddlers. In contrast to the findings of the present study, higher levels of paternal [83] or maternal physical activity [84, 85], and higher perceived safety of outdoor play environments [55] have also been found to be associated with higher physical activity among toddlers in previous studies. It is difficult to determine the most important correlates associated with toddlers’ physical activity at this time because of the dearth of empirically based knowledge in this age group. Further research is needed to confirm our findings as well as explore whether other correlates not examined in this study or understudied in previous literature (e.g., co-participation, built environment, child care environment) are of importance, and whether other theoretical models are better suited to guide the examination of correlates of toddlers’ physical activity.

The major strengths of this study are the relatively large sample of toddlers and the objective measure of physical activity. The use of structural equation modeling with the application of Bayes’ theorem, which allowed us to simultaneously evaluate the associations among multiple important variables assessed via the hypothesized model is another strength. Furthermore, these methods are known to improve estimation accuracy in complex statistical models such as structural equation modeling under cases of small sample sizes. In addition, Bayesian methods using MCMC allow structural equation models to have binary outcome variables. Other study strengths include the theoretical underpinning and the demographically and socioeconomically diverse sample. Lastly, this study included behavior-specific correlates for physical activity and screen time within the same sample.

A limitation of this study is the use of proxy-reported data except for toddlers’ physical activity; thus, our findings are subject to information bias (e.g., recall and social desirability). Nonetheless, psychometric properties of the PREPS questionnaire are previously established [19]. The cross-sectional design is a further limitation of this study. However, our hypotheses and analyses were guided by a theory and developed based upon temporal directionality and model specification [86]. Only 64% of the analytic sample had accelerometry data; thus, the physical activity specific analyses included significantly fewer toddlers compared to the screen time specific analyses. However, no demographic differences were observed between included and excluded samples. In addition, some studies have suggested that the correlates of physical activity and screen time may differ by sex [87], ethnicity [88], and socioeconomic status [89] as early as the preschool years. Though we were not able to test sociodemographic-stratified models, sociodemographic correlates of physical activity and screen time identified in a previous study involving the same sample [28] were controlled in the SEM analysis.

## Conclusions

Parents appear to play a critical role in establishing healthy screen time behaviors among their toddlers by practicing screen time limits, role modeling healthy screen time behaviors themselves, and creating screen-free bedrooms within the home environment. However, important correlates of physical activity in toddlers are currently unclear. Further investigation is required to build on our findings with regard to the potential role of parental and environmental characteristics in toddlers’ screen time using longitudinal and experimental study designs to confirm causality. Given that toddlerhood is a window of opportunity for establishing healthy behavioral patterns at home, and that the design of our hypothesized model is guided by the SMCB model, our results can help inform future family-based interventions targeting screen time among toddlers.

## Appendix

**Table:** **Table 4**

	Standardized direct effect	Standard deviations	95% Credible Intervals	Convergence
Figure 2				
Posterior predictive *p* = 0.48				
Neighborhood safety - > Parental limits	− 0.008	0.067	−0.138, 0.125	1.001
Neighborhood safety - > Parental modeling	−0.135	0.073	−0.279, 0.012	1.001
Neighborhood safety - > Toddler’s screen time	−0.079	0.061	−0.199, 0.042	1.001
Negative outcome expectations - > Parental modeling	0.034	0.081	−0.127, 0.212	1.000
Negative outcome expectations - > Parental limits	**−0.147**	0.070	−0.169, − 0.125	1.000
Negative outcome expectations - > Electronics in bedroom	0.092	0.078	−0.058, 0.248	1.001
Negative outcome expectations - > Toddler’s screen time	0.087	0.066	−0.043, 0.217	1.001
Barrier self-efficacy - > Parental limits	**0.451**	0.065	0.315, 0.572	1.001
Barrier self-efficacy - > Parental modeling	−0.108	0.080	−0.269, 0.052	1.000
Barrier self-efficacy - > Electronics in bedroom	−0.132	0.082	−0.292, 0.029	1.001
Barrier self-efficacy - > Toddler’s screen time	−0.047	0.073	−0.187, 0.097	1.001
Parental limits - > Electronics in bedroom	0.058	0.069	−0.095, 0.211	1.001
Parental limits - > Toddler’s screen time	**−0.179**	0.066	−0.309, − 0.052	1.000
Parental modeling - > Electronics in bedroom	**0.146**	0.068	0.010, 0.277	1.000
Parental modeling - > Toddler’s screen time	**0.212**	0.058	0.099, 0.325	1.001
Electronics in bedroom - > Toddler’s screen time	**0.250**	0.059	0.130, 0.365	1.001

## Abbreviations

CI: credible interval
ICC: intraclass correlation
LPA: light-intensity physical activity
M: mean
MCMC: Markov Chain Monte Carlo
MET: metabolic equivalent of tasks
MVPA: moderate- to vigorous-intensity physical activity
PREPS: Parents’ Role in Establishing healthy Physical activity and Sedentary behavior habits
SD: standard deviation
SEM: structural equation modeling
SMCB: Socialization Model of Child Behavior
TPA: total physical activity
TV: television
VG: video game
